# The immune landscape of fetal chorionic villous tissue in term placenta

**DOI:** 10.3389/fimmu.2024.1506305

**Published:** 2025-01-13

**Authors:** Brianna M. Doratt, Heather E. True, Suhas Sureshchandra, Qi Qiao, Monica Rincon, Nicole E. Marshall, Ilhem Messaoudi

**Affiliations:** ^1^ Department of Microbiology, Immunology and Molecular Genetics, University of Kentucky, Lexington, KY, United States; ^2^ Department of Pharmaceutical Sciences, University of Kentucky, Lexington, KY, United States; ^3^ Department of Physiology and Biophysics, School of Medicine, University of California, Irvine, Irvine, CA, United States; ^4^ Institute for Immunology, University of California, Irvine, Irvine, CA, United States; ^5^ Department of Biostatistics, University of Kentucky, Lexington, KY, United States; ^6^ Maternal-Fetal Medicine, Oregon Health and Science University, Portland, OR, United States

**Keywords:** placenta, transcriptomics, epigenomics, monocyte, macrophage, pregnancy, obesity, microglia

## Abstract

**Introduction:**

The immune compartment within fetal chorionic villi is comprised of fetal Hofbauer cells (HBC) and invading placenta-associated maternal monocytes and macrophages (PAMM). Recent studies have characterized the transcriptional profile of the first trimester (T1) placenta; however, the phenotypic and functional diversity of chorionic villous immune cells at term (T3) remain poorly understood.

**Methods:**

To address this knowledge gap, immune cells from human chorionic villous tissues obtained from full-term, uncomplicated pregnancies were deeply phenotyped using a combination of flow cytometry, single-cell RNA sequencing (scRNA-seq, CITE-seq) and chromatin accessibility profiling (snATAC-seq).

**Results:**

Our results indicate that, relative to the first trimester, the frequency of fetal macrophages (HBC, proliferating HBC) is significantly reduced, whereas that of infiltrating maternal monocytes/macrophages (PAMM1b, PAMM1a, PAMM2, MAC_1) increased in T3. PAMM1b and HBCs exhibit the most phagocytic capacity at term highlighting their regulatory role in tissue homeostasis in late pregnancy. The transcriptional profiles of resident villous immune subsets exhibit a heightened activation state relative to the relative to T1, likely to support labor and parturition. Additionally, we provide one of the first insights into the chromatin accessibility profile of villous myeloid cells at term. We next stratified our findings by pre-pregnancy BMI since maternal pregravid obesity is associated with several adverse pregnancy outcomes. Pregravid obesity increased inflammatory gene expression, particularly among HBC and PAMM1a subsets, but dampened the expression of antimicrobial genes, supporting a tolerant-like phenotype of chorionic villous myeloid cells. We report a decline in HBC abundance accompanied by an increase in infiltrating maternal macrophages, which aligns with reports of heightened chorionic villous inflammatory pathologies with pregravid obesity. Finally, given the shared fetal yolk-sac origin of HBCs and microglia, we leveraged an *in vitro* model of umbilical cord blood-derived microglia to investigate the impact of pregravid obesity on fetal neurodevelopment. Our findings reveal increased expression of activation markers albeit dampened phagocytic capacity in microglia with pregravid obesity.

**Discussion:**

Overall, our study highlights immune adaptations in the fetal chorionic villous with gestational age and pregravid obesity, as well as insight towards microglia dysfunction possibly underlying poor neurodevelopmental outcomes in offspring of women with pregravid obesity.

## Introduction

The placenta harbors a unique immune landscape that undergoes carefully orchestrated changes to promote fetal growth and tolerance, debris clearance, and antimicrobial defense (1). While the maternal decidua is composed mostly of decidual natural killer cells (dNKs), macrophages (dMac), and T cells (2–4), the immune compartment within fetal chorionic villi is comprised exclusively of myeloid cells, including fetal Hofbauer cells (HBC) and heterogenous populations of invading placenta-associated maternal monocytes and macrophages (PAMM) (4–7). HBCs originate in the fetal yolk sac and participate in antimicrobial responses, placental angiogenesis, and remodeling (5, 6, 8–10). Akin to HBCs, brain resident macrophages called microglia are also derived from the fetal yolk sac (10). As such, recent studies using rodent models have highlighted the potential role of placental tissues and immune cells in modeling fetal neurodevelopment (11) but have yet to be described using human samples.

The more recently described PAMM subsets aid in tissue repair (4–6) and regulation of immune responses (6). PAMM1a cells are the most abundant PAMM subset in the first trimester and localize to the placental surface, where they regulate placental development and repair. PAMM1b cells are morphologically similar to peripheral blood monocytes and secrete elevated amounts of pro-inflammatory cytokines (IL-1β, IL-6) compared to PAMM1a. Finally, the PAMM2 subset is found in very low abundance in early pregnancy and is believed to be contaminating maternal macrophages from the decidual compartment (4, 5). While recent studies have characterized the functional and phenotypic diversity of immune cells in the placenta during early gestation (4, 12, 13), the phenotypic and functional diversity of immune cells in the chorionic villous at term remains less clear (14, 15).

Additionally, nearly one-third of pregnancies in the United States are impacted by pre-pregnancy (pregravid) obesity (16) which has been associated with increased rates of abnormal placental pathologies (17–19) as well as several obstetric and post-partum complications (20–23) including early pregnancy loss and preterm delivery (24–26), stillbirth (20, 27), large-for-gestational-age infants (24, 28), and cognitive impairment (9, 29). These adverse outcomes are thought to be due to altered fetoplacental immune parameters, notably chronic villitis (30, 31), decreased expression of HLA-G required for immune tolerance (32), and increased macrophage migration into placental villous tissue (18, 33). The dysregulation of placental macrophages has been linked with many placental pathologies and obstetric complications (5, 34–43). Indeed, HBC morphology and function are altered by maternal obesity, including increased density and size (39, 44) and heightened responses to LPS stimulation (9, 10) potentially mediated by an influx of inflammatory CD68+ HBCs (45). However, current studies have not addressed longitudinal changes in HBC or PAMM phenotypes and function with pregravid obesity.

To address these knowledge gaps, we first deeply phenotyped immune cells within chorionic villi using a combination of flow cytometry, single-cell RNA sequencing (scRNA-seq), cellular indexing of transcriptomes and epitopes sequencing (CITE-seq), single-nuclei ATAC sequencing (snATAC-seq), and functional assays. We then compared our findings to those obtained from the first-trimester placenta (6). Next, we stratified our findings in the third-trimester placenta by maternal Body Mass Index (BMI) to interrogate the impact of maternal obesity on the term chorionic villous immune landscape. Finally, we leveraged an established model of microglia-like cells derived from umbilical cord blood (45) of newborns from lean pregnancies compared to those with obesity to better understand the implications of pregravid obesity on fetal neurodevelopment (9, 10).

## Materials and methods

### Ethics approval statement

This study was approved by the Institutional Ethics Review Board of Oregon Health & Science University and the University of California, Irvine.

### Cohort characteristics

A total of 46 non-smoking women who had an uncomplicated pregnancy were enrolled in this study (**Table 1**). For experiments stratified by pregravid obesity, participants were categorized by their pre-pregnancy body mass index (BMI) as lean (N=24, BMI >20 and <25 kg/m^2^) or with obesity (N=22, BMI >30kg/m^2^). All subjects underwent scheduled cesarean deliveries for a variety of medical and elective indications, including repeat cesarean, breach presentation, maternal request, and medical contraindication to vaginal birth. Exclusion criteria include active maternal infection, documented fetal congenital anomalies, substance use disorder, chronic illness requiring regular medication use, preeclampsia, gestational diabetes, chorioamnionitis, and significant medical conditions (active cancers, cardiac, renal, hepatic, or pulmonary diseases).

**Table 1:** Cohort characteristics.

	Lean	Obese
Enrollment	24	22
BMI (kg/m^2^) *	21.61 ± 2.10	37.51 ± 6.36
Maternal Age (years)	34.42 ± 5.27	30.95 ± 4.57
Gestational Age (Days)	271.33 ± 4.10	267.45 ± 6.37
% Female	50	41

Avg (SD). *p<=0.0001.

### Sample collection and processing

Placental biopsies were processed and leukocytes isolated as previously described (46). Umbilical cord mononuclear cells (UCBMC) were obtained by standard density gradient centrifugation over Lymphoprep density gradient medium (STEMCell Technologies, Cambridge, MA) (47). Cells were resuspended in 10% DMSO/FBS, frozen using Mr. Frosty Freezing Containers (Thermo Fisher Scientific,Waltham, MA), and stored in liquid nitrogen until thawed and plated for induction to microglia-like cells.

### CD45 3’ single cell RNA library preparation

Immune cells from chorionic villi, decidua, maternal and cord blood (N=4, 2 lean and 2 obese) were thawed, stained with CD45-FITC at 4°C in 1% FBS in DPBS without calcium and magnesium and sorted on BD FACS Aria Fusion into RPMI (supplemented with 30% FBS) using SYTOX Blue stain for dead cell exclusion. Cells were counted in triplicate on a TC20 Automated Cell Counter (BioRad, Hercules, CA). An equivalent number of cells were pooled by group (lean and obese) and resuspended in DPBS with 0.04% BSA in a final concentration of 1200 cells/μL, and immediately loaded on the 10x Genomics Chromium Controller with a loading target of 17,600 cells. Libraries were generated using the V3 chemistry per the manufacturer’s instructions (10x Genomics, Pleasanton, CA). Libraries were sequenced on Illumina NovaSeq with a sequencing target of 50,000 reads per cell.

### TotalSeq (CITEseq) library preparation

Chorionic villous leukocytes (N=8, 4 lean and 4 obese) were thawed, washed, filtered, and stained with Ghost Violet 450 (Tonbo Biosciences, San Diego, CA) for 30 min in the dark at 4°C. Samples were washed with a cell staining buffer (DPBS with 0.5% BSA), Fc blocked for 10 min (Human TruStain FcX, Biolegend, San Diego, CA), incubated with a cocktail containing CD14 (FITC) and CCR2 (APC) and 0.5 μg of each oligo tagged antibody; CD9 (TotalSeq™-B0579), FOLR2 (TotalSeq™-B0427), HLA-DR (TotalSeq™-B0159) and CD62L (TotalSeq™-B0147) (Biolegend, San Diego, CA), for 30 min at 4°C. Samples were washed four times with 1X PBS, filtered using Flowmi 1000 μL pipette strainers (SP Bel-Art, Wayne, NJ), and resuspended in 300 μL FACS buffer.

CD14+CCR2+/- cells were sorted on the BD FACS Aria Fusion and counted in triplicates on a TC20 Automated Cell Counter (BioRad, Hercules, CA). An equivalent number of cells were pooled by group (lean and obese) and resuspended in DPBS with 0.04% BSA in a final concentration of 1500 cells/μL. Single-cell suspensions were immediately loaded on the 10x Genomics Chromium Controller with a loading target of 20,000 cells. Libraries were generated using the 3’ (V3.1) and Feature Barcode Library Kit per manufacturer’s instructions (10X Genomics, Pleasanton CA). Libraries were sequenced on Illumina NovaSeq with a sequencing target of 30,000 reads per cell for gene expression libraries and 10,000 reads per cell for CITEseq.

### Single cell RNA sequencing analysis and data integration

For 3’ Single Cell RNAseq library data preprocessing, raw reads were aligned and quantified using the CellRanger Software Suite (Version 3.0.1 10X Genomics, Pleasanton, CA) against the GRCh38 human reference genome. Downstream processing of aligned reads was performed using Seurat (Version 3.1.5). Droplets with ambient RNA or potential doublets (<400 or >4000 detected genes) and dying cells (>20% total mitochondrial gene expression) were excluded during initial QC. Data objects for the chorionic villous, decidua, maternal, and cord blood were integrated as described previously to remove infiltrating leukocytes from the chorionic villous resident cells (46).

For first-trimester single cell RNA library data preprocessing, raw sequencing files from single cell RNAseq libraries generated by the Vento-Tormo lab from (4) total placental digest were downloaded from ArrayExpress (E-MTAB-7304XX). Raw reads were aligned and quantified as outlined above. Cleaned chorionic villous leukocytes from the CD45 3’, Totalseq and first-trimester data were integrated using Seurat’s *IntegrateData* function. Data normalization and variance stabilization were performed on the integrated object using the *NormalizeData* and *ScaleData* functions in Seurat, where a regularized negative binomial regression was corrected for differential effects of mitochondrial and ribosomal gene expression levels. Dimensional reduction was performed using the *RunPCA* function to obtain the first 30 principal components and clusters visualized using Seurat’s *RunUMAP* function. Cell types were assigned to individual clusters using *FindAllMarkers* function with a log2 fold change cutoff of at least 0.4, FDR<0.05, and using a known catalog of well-characterized scRNA markers for human chorionic villous leukocytes (**Supplementary Table 1**) (4). Differential gene expression analysis was performed using *MAST* function in Seurat using an FDR<0.05 and a log2 fold change ± 0.4. Functional enrichment was performed using Metascape (48). Module scores for specific pathways/gene sets were incorporated cluster-wise using the *AddModuleScores* function (**Supplementary Table 2**).

### Derivation of microglia-like cells from UCBMC

Derivation of microglia-like cells from UCBMC was adapted from the protocol previously published by Sheridan et al. (45). Briefly, frozen UCBMC were thawed and 1x10^6^ cells were plated per well of a 24-well TC plate coated with poly D-lysine (50μg/ml). 500μl of media containing RPMI 1640, 10% FBS, and 1% penicillin/streptomycin was added to each well and plates placed in 37°C incubator with 5% CO_2_ for 24 hours. After 24 hours, the media was replaced with induction media containing RPMI 1640, 1% Glutamax, 1% penicillin/streptomycin, 10μg IL-34, and 5μg GM-CSF and plate returned to 37°C incubator with 5% CO_2_ for an additional 12 days. On day 13 post seeding, media was replaced with fresh induction media. iMGL were harvested on day 14 post seeding for downstream experiments.

### Phenotyping by flow cytometry

Chorionic villous leukocytes (N=19, 10 lean and 9 obese) were thoroughly washed with FACS buffer and stained with a cocktail of the following surface antibodies: CD45, CD14, HLA-DR, FOLR2, CD9, CCR2, CD62L, CD163, CD86, CD64, and CD11c (BioLegend, San Diego, CA). To prevent non-specific binding by myeloid cells, True-Stain Monocyte Block and Human TruStain FcX™ (Fc Receptor Blocking Solution, BioLegend, San Diego, CA) were also added to the surface staining cocktail (1:20). After incubation for 30 minutes at 4°C, cell pellets were washed with FACS buffer, and were run using an Attune NxT and analyzed on FlowJo 10.10 (Beckton Dickinson, Ashland, OR).

iMGLs (N=10, 5 lean and 5 obese) were thoroughly washed with FACS buffer and stained with a cocktail of the following surface antibodies at a ratio of 1:20: CD14, HLA-DR, CD16, CX3CR1, CD45, CD11b, P2RY12, TMEM119, TREM2, CD68, CD40, CD163, CD115, and CD86 (BioLegend, San Diego, CA). To prevent non-specific binding by myeloid cells, True-Stain Monocyte Block and Human TruStain FcX™ (Fc Receptor Blocking Solution) (BioLegend, San Diego, CA) were also added to the surface staining cocktail (1:20). After incubation for 30 minutes at 4°C, the iMGL cell pellet was washed with FACS buffer, fixed for 20 minutes (Tonbo fix/permeabilization solution, 1:3), permeabilized (Tonbo permeabilization buffer), and stained with the following panel of intranuclear antibodies: PU.1, IBA.1, and IRF8 (BioLegend, San Diego, CA) at a ratio of 1:20 overnight at 4°C. The next day, stained iMGL were washed with FACS buffer, and were run using an Attune NxT and analyzed on FlowJo 10.10 (Beckton Dickinson, Ashland, OR).

### Phagocytosis assay

Chorionic villous leukocytes (N=20, 10 lean and 10 obese) or iMGL (N=10, 5 lean and 5 obese) were incubated for 2 hours at 37°C in media containing 1 mg/mL pH-sensitive pHrodo *E. coli* BioParticles conjugates (ThermoFisher Scientific, Waltham, MA). Pellets were washed twice, surface stained with antibodies at a ratio of 1:20 against CD14, HLA-DR, FOLR2, CD9, and CCR2 (for chorionic villous leukocytes) or CD2, CD20, CD45, CD14, HLA-DR, P2RY12 (for iMGL), (BioLegend, San Diego, CA), then resuspended in ice cold FACS buffer. Samples were run using an Attune NxT and analyzed on FlowJo 10.10 (Beckton Dickinson, Ashland, OR).

### Ex vivo stimulation and intracellular cytokine staining

To measure cytokine responses, 1x10^6^ thawed leukocytes (N=6 for chorionic villi, N=9-10 for decidua) were stimulated for 16 hours at 37°C in RPMI supplemented with 10% FBS in the presence or absence of bacterial TLR cocktail containing 1mg/mL LPS-B5 (TLR4 ligand, *E.coli* 055:B5; cat#: tlrl-b5lps, *In vivo*gen, San Diego, CA), 2mg/mL Pam3CSK4 (TLR1/2 agonist, cat#:TLRL-PMS, *In vivo*gen, San Diego, CA), and 1mg/mL FSL-1 (TLR2/6 agonist, cat#:SML1420, Sigma, St. Louis, MO). Brefeldin A (Biolegend, San Diego, CA) was added after 1 hour incubation and cells were cultured for an additional 15 hours at 37°C before surface and intracellular staining.

To delineate HBC and PAMM subsets, villous leukocytes were stained with the following surface antibodies at a ratio of 1:20: CD45, CD14, HLA-DR, FOLR2, CD9 and CCR2 (BioLegend, San Diego, CA) for 30 minutes in the dark at 4°C. Samples were fixed and permeabilized using fixation and permeabilization wash buffer (BioLegend, San Diego, CA) at 4°C for 20 minutes and stained intracellularly for 4 hours for TNFα and IL-6 (BioLegend, San Diego, CA) at a ratio of 1:20. Decidual leukocytes were stained with the following surface antibodies at a ratio of 1:20: CD45, CD2, CD20, CD14, HLA-DR, CD11c, CD9, and CCR2 (BioLegend, San Diego, CA) for 30 minutes in the dark at 4°C. Samples were fixed, permeabilized, and stained intracellularly as outlined above for villous cells. Cells were then washed with FACS buffer and acquired using the Attune NxT Flow Cytometer (ThermoFisher Scientific, Waltham MA) and analyzed using FlowJo 10.10 (Beckton Dickinson, Ashland OR).

### Luminex assay

Flash frozen chorionic villous tissue (N=19/group) were homogenized by adding 200mg of sample to a 2ml starstedt tube containing silica carbide beads and placed in bead beater for 3 cycles of 10 seconds each. Homogenized chorionic villous was then centrifuged, supernatant collected into a new Eppendorf tube, centrifuged a second time to remove particulates, and supernatant collected for Luminex assay per manufacturer’s instructions (R&D Human Luminex^®^ Discovery Assay, 29-plex, Catalog #: LXSAHM).

### Single-nuclei ATAC-seq library preparation

Chorionic villous leukocytes (n=8, 4 lean, 4 obese) were thawed, and surface stained with CD14, HLA-DR, FOLR2, CD9, CCR2 (1:20) and Sytox Green (1:100) then sorted on a BD FACSAria Fusion (**Supplementary Figure 3B**). Nuclei were isolated from each subset, counted, and transposition reaction performed according to manufacturer’s instructions (10X Genomics). ~10,000 nuclei were obtained per sample, except for PAMM2 where ~1000 nuclei were obtained and loaded into the 10X Chromium Controller. Library preparation (10X v2 chemistry) was performed for samples according to manufacturer protocol and sequenced on a NovaSeq S4 (Illumina) to a depth of >25,000 paired reads/cell.

### Single-nuclei ATAC-seq data analysis

Sequencing reads were pre-processed using the cellranger-atac pipeline (v2.1.0) (10X Genomics) where accessibility counts for each cell were aligned to the GRCh38 reference genome. The ArchR package (47) (v1.0.1) was used for downstream analysis following their vignette in R (v4.1.1). Arrow files were created from each sample fragment file and low-quality cells were filtered out (<1000 fragments, <4 TSS enrichment, doublets calculated by addDoubletScores). An ArchR project was created by combining all Arrow files. Iterative latent semantic indexing (LSI) was performed as the first dimensional reduction followed by the *addHarmony* function to correct batch effects. Uniform manifold approximation and projection (UMAP) was used for the final dimensional reduction and clusters were added using the *addClusters* function. Marker features for each cluster based on gene scores were identified using the *getMarkerFeatures* function (**Supplementary Table 3**). Pseudo-bulk replicates were created for peak calling using MACS2. Per-cell deviations across motif annotations were computed using the *addDeviationsMatrix* function. The *plotBrowserTrack* function was used to generate the track plotting with ArchRBrowser. All UMAP and heatmap plots were generated using ArchR functions. Genomic annotation of open chromatin regions related to the promoter, 5’ UTR, downstream, and distal intergenic regions in DAR analysis was assigned using ChIPSeeker. Promoters were defined as: −1000 bp to +100 bp around the transcriptional start site (TSS). Genes with no annotations were excluded from downstream analyses. Functional enrichment analysis of the promoters and intergenic regions was performed using Metascape (48).

### Statistical analyses

All statistical analyses were conducted in Prism 10 (GraphPad). All definitive outliers in two-way and four-way comparisons were identified using ROUT analysis (Q=0.1%) after testing for normality using Shapiro-Wilk test (alpha=0.05). If data were normally distributed across all groups, differences were tested using ordinary one-way ANOVA with unmatched samples. Multiple comparisons were corrected using Holm-Sidak test adjusting the family-wise significance and confidence level at 0.05. If the Gaussian assumption was not satisfied, differences were tested using the Kruskall-Wallis test (alpha=0.05) followed by Dunn’s multiple hypothesis correction tests. Differences in normally distributed two groups were tested using an unpaired t-test with Welch’s correction (assuming different standard deviations). Two group comparisons that failed normality tests were tested for differences using Mann-Whitney test.

## Results

### The integrated immune landscape of the chorionic villous

**Figure 1:**
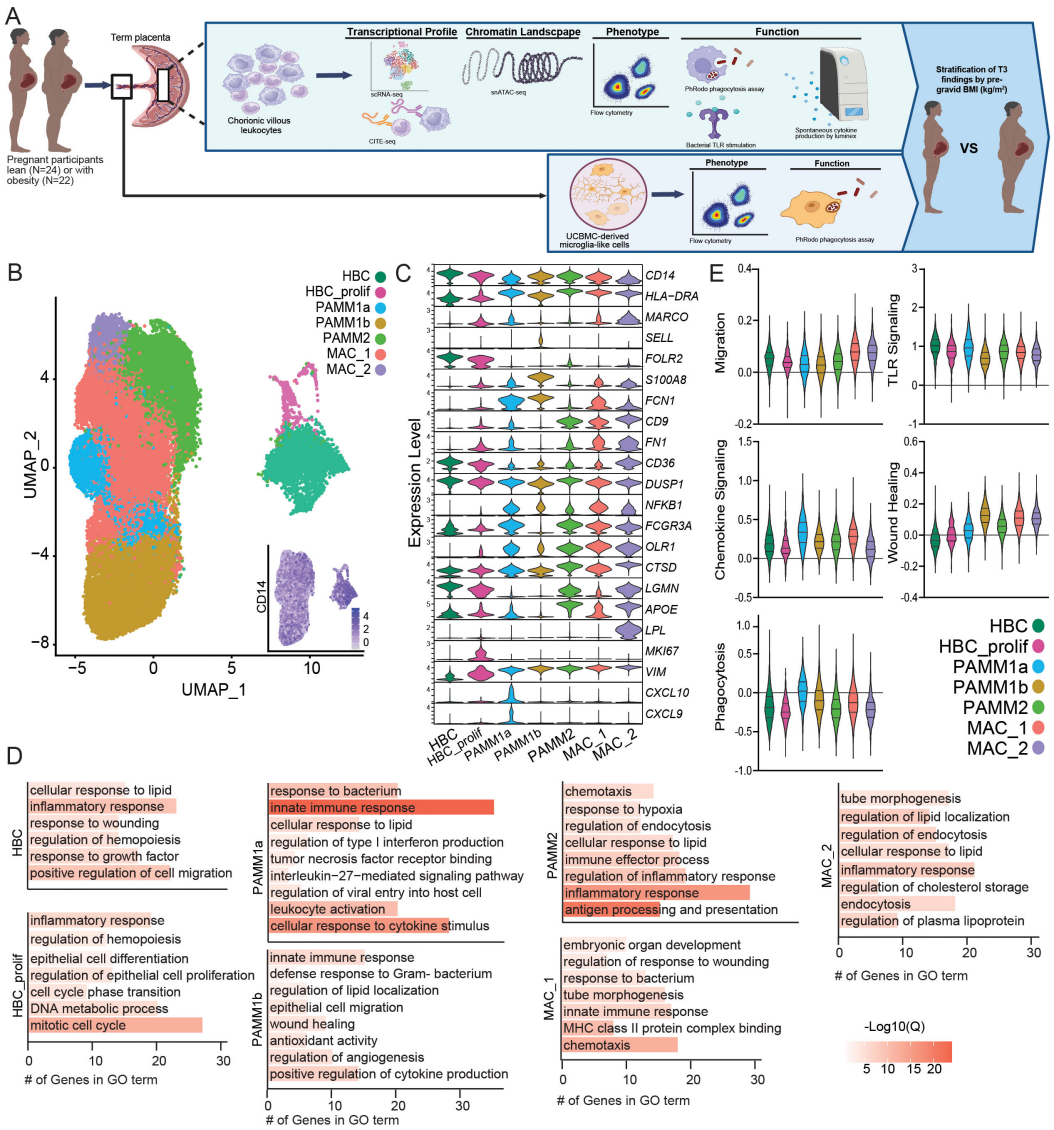
Integration of first trimester and term chorionic villous transcriptional profiles. **(A)** Overarching experimental design – leukocytes were isolated from chorionic villous tissues from term cesarean deliveries (N=46 total, 24 lean and 22 obese). Chorionic villous leukocytes were FACS sorted and subjected to gene expression profiling using 10x 3’single-cell gene expression protocol (scRNA-seq), transcriptome-based profiling using CITE-seq, or snATAC-seq for chromatin accessibility. Expression of activation markers, phagocytic capacity, and responses to bacterial TLR ligands by total chorionic villous leukocytes were assayed using flow cytometry. Spontaneous cytokine production by chorionic villous leukocytes was measured by Luminex assay. Finally, microglia-like cells were derived from UCBMC (iMGL) and expression of activation markers and phagocytic capacity of iMGLs were assayed using flow cytometry. **(B)** Uniform Manifold Approximation and Projection (UMAP) representation of integrated data with 36,598 leukocytes with an embedded feature plot of the expression of *CD14.* **(C)** Violin plots showing expression levels of markers genes used for cluster identification. **(D)** Bar graph of gene ontology (GO) terms associated with cluster marker genes. Length of the bar indicates the number of genes associated with the GO term and the color indicates log10(Q-value). **(E)** Violin plots showing module scores for the indicated pathways for each cell cluster. All comparisons between chorionic villous subsets were statistically significant (p<0.05).

Pregnant women undergoing scheduled cesarean delivery at term (>37 weeks’ gestation) with otherwise uncomplicated pregnancies were classified based on pregravid body mass index (BMI) into lean participants (BMI >20 and <25 kg/m^2^) or those with obesity (BMI >30 kg/m^2^) (**Table 1**, **Figure 1A**), since previous studies have shown that pregravid BMI strongly correlates with fat mass (49). Fetal chorionic villous tissues were separated from maternal decidual tissues, digested, and then subjected to Percoll gradient centrifugation to obtain leukocytes. To deeply characterize the myeloid compartment within fetal chorionic villi in an unbiased manner, we began by profiling the CD45^+^ (total immune) compartment using single-cell RNA sequencing (scRNA-seq) (N=4) (**Supplementary Figure 1A**). To eliminate the possibility of contaminating maternal blood and decidual cells in chorionic villi, we integrated this dataset with matched maternal blood and decidua and autologous umbilical cord blood mononuclear cells (**Supplementary Figure 1B**) (46) and excluded overlapping clusters from subsequent analysis. This dataset was integrated with scRNA-seq on FACS-sorted myeloid cells (CD14^+^CCR2^+/-^) in an independent set of chorionic villi (N=8). Finally, these data were integrated with myeloid cells from early gestation (week 14) recently described by Vento-Tormo et al. (4), after clustering and exclusion of T cells, B cells, NK cells, and non-immune compartments of the early placenta (**Supplementary Figures 1A, C**). All three datasets showed significant overlap (**Supplementary Figure 1D**).

A distinct population of HBCs expressing *FOLR2* without *HLA-DR* and a population of proliferating HBCs expressing *MKI67* were identified (**Figures 1B, C**). The PAMM1 subsets were identified based on *HLA-DR^high^FOLR2^low^* expression and further subdivided into PAMM1a (*CD9^high^CXCL9/10^high^*) and PAMM1b (*CD9^low/-^*) (**Figures 1B, C**). The PAMM2 population was identified based on *HLADR^high^FOLR2^high^CD9^high^* expression (**Figures 1B, C**). Finally, two additional macrophage populations, MAC_1 (*HLADR^high^FN1^high^NFKB^high^LPL^low^*) and MAC_2 (*HLADR^high^CD9^high^LPL^high^*), with distinct expression profiles from PAMM and HBC populations were identified (**Figures 1B, C**).

Next, we performed functional enrichment of the marker genes of each monocyte/macrophage cluster to uncover their functional potential. Transcriptional signatures of HBC, PAMM1b, and MAC_1 subset enriched for wound healing and cell migration/chemotaxis (**Figure 1D**). The proliferating HBC cluster additionally expressed high levels of genes important for mitosis and DNA metabolism (**Figure 1D**). Furthermore, marker genes of PAMM1a, PAMM1b, PAMM2, and MAC_1 clusters enriched to gene ontology (GO) terms linked to innate immunity, leukocyte activation, and anti-microbial responses (**Figure 1D**). Marker genes for the PAMM2 cluster also enriched to processes implicated in antigen processing and presentation as well as responses to hypoxia, lipids, and inflammation (**Figure 1D**). Finally, the MAC_1 and MAC_2 cluster marker genes mapped to signatures associated with fetal development (**Figure 1D**), with MAC_1 cluster marker genes also enriching to anti-microbial responses, whereas the MAC_2 cluster marker genes uniquely mapped to lipid storage and regulation (**Figure 1D**).

We next measured module scores related to myeloid cell function - migration, chemokine signaling, phagocytosis, TLR signaling, and wound healing – were all significantly different across subsets (**Figure 1E**). MAC_1 and MAC_2 expressed the highest migration and wound healing module scores, while the PAMM1a subset expressed higher module scores of TLR and cytokine/chemokine signaling as well as phagocytosis. Interestingly, phagocytosis and wound healing module scores were low within the HBCs subsets (**Figure 1E**).

### Comparison of the immune landscape of the chorionic villous at T1 and term

**Figure 2:**
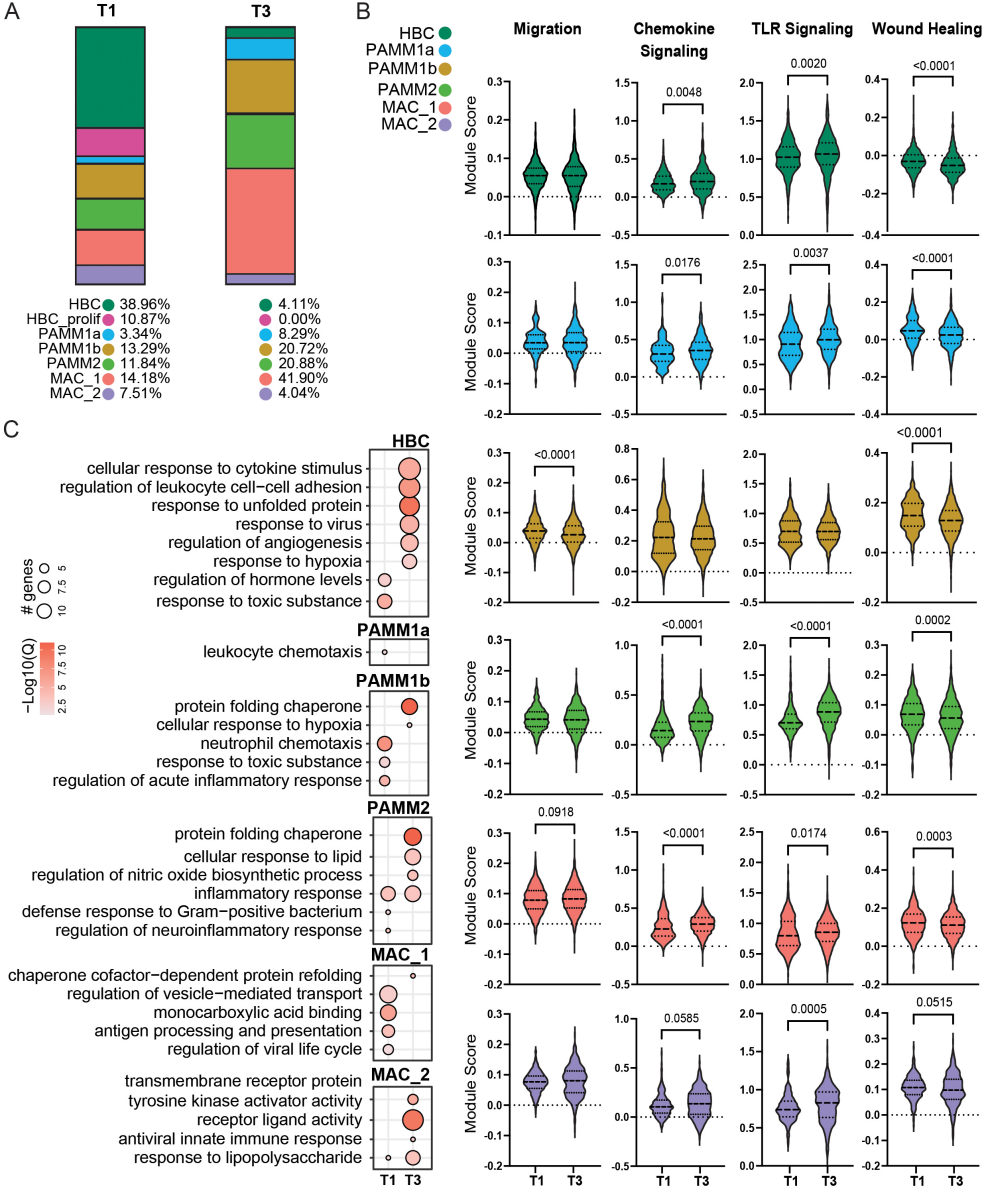
Comparison of T1 and T3 chorionic villous immune landscape. **(A)** Stacked bar graphs of cluster frequencies for T1 (left) and T3 (right) chorionic villous from scRNA-seq data. **(B)** Violin plots of module scores for the indicated terms in T1 and T3. **(C)** Bubble plot of select GO terms for DEGs upregulated in T1 or T3 for each cell cluster. The size of the bubble denotes the number of genes mapping to each gene ontology (GO) term, and the intensity of color denotes the -log10(Q-value).

The chorionic villous undergoes dramatic changes with gestational age to support fetal growth and development. To test if these changes were associated with phenotypic and functional changes within the myeloid compartment, we compared our findings at term to those previously reported at T1 (**Supplementary Figure 1A**). Comparison between T1 and T3 revealed a decrease in the frequency of HBC, proliferating HBC, and MAC_2 subsets at term (**Figure 2A**). On the other hand, the relative abundance of all PAMM subsets and MAC_1 increased with gestational age (**Figure 2A**). Next, we compared module scores related to macrophage effector functions between T1 and T3 (**Figure 2B**). Aggregate expression of genes in chemokine and TLR signaling modules increased with gestation, whereas those related to wound healing were decreased at T3 in most subsets (**Figure 2B**). Signatures associated with cell migration were reduced in the PAMM1b cluster (**Figure 2B**).

Differential gene expression analyses revealed activation of pathways associated with response to cytokines, hypoxia, and viruses but downregulation of hormone regulation pathways in HBC at T3 relative to T1 (**Figure 2C**). An upregulation of genes important for leukocyte chemotaxis was noted at T1 for PAMM1a (**Figure 2C**). Genes more highly expressed at T1 by PAMM1b cells enriched to GO terms associated with inflammatory responses and neutrophil recruitment (**Figure 2C**). In contrast, genes upregulated in T3 PAMM1b cells were associated with hypoxia and protein homeostasis (**Figure 2C**). Within the PAMM2 cluster, genes important for anti-bacterial responses and neuroinflammation were more highly expressed in T1. In contrast, those important for lipids and oxidative stress responses were more highly expressed in T3 (**Figure 2C**). Genes important for antigen processing and presentation, response to virus, and vesicle transport were expressed at higher levels at T1 in MAC_1 and those important for protein folding at T3 in MAC_1 (**Figure 2C**). Finally, increased expression of genes associated with response to LPS, antiviral responses, and cell signaling were prevalent at T3 in the MAC_2 cluster (**Figure 2C**). Collectively, these data indicate that while the frequencies of resting and proliferating HBCs are reduced with gestational age, they exhibit features of enhanced activation and cytokine signaling at term. Additionally, PAMM and MAC_2 subsets increase in abundance with gestational age, but express genes associated with tissue homeostasis, suggesting a more regulatory role of these subsets in late pregnancy.

### Functional responses of myeloid cells in the term chorionic villous

**Figure 3:**
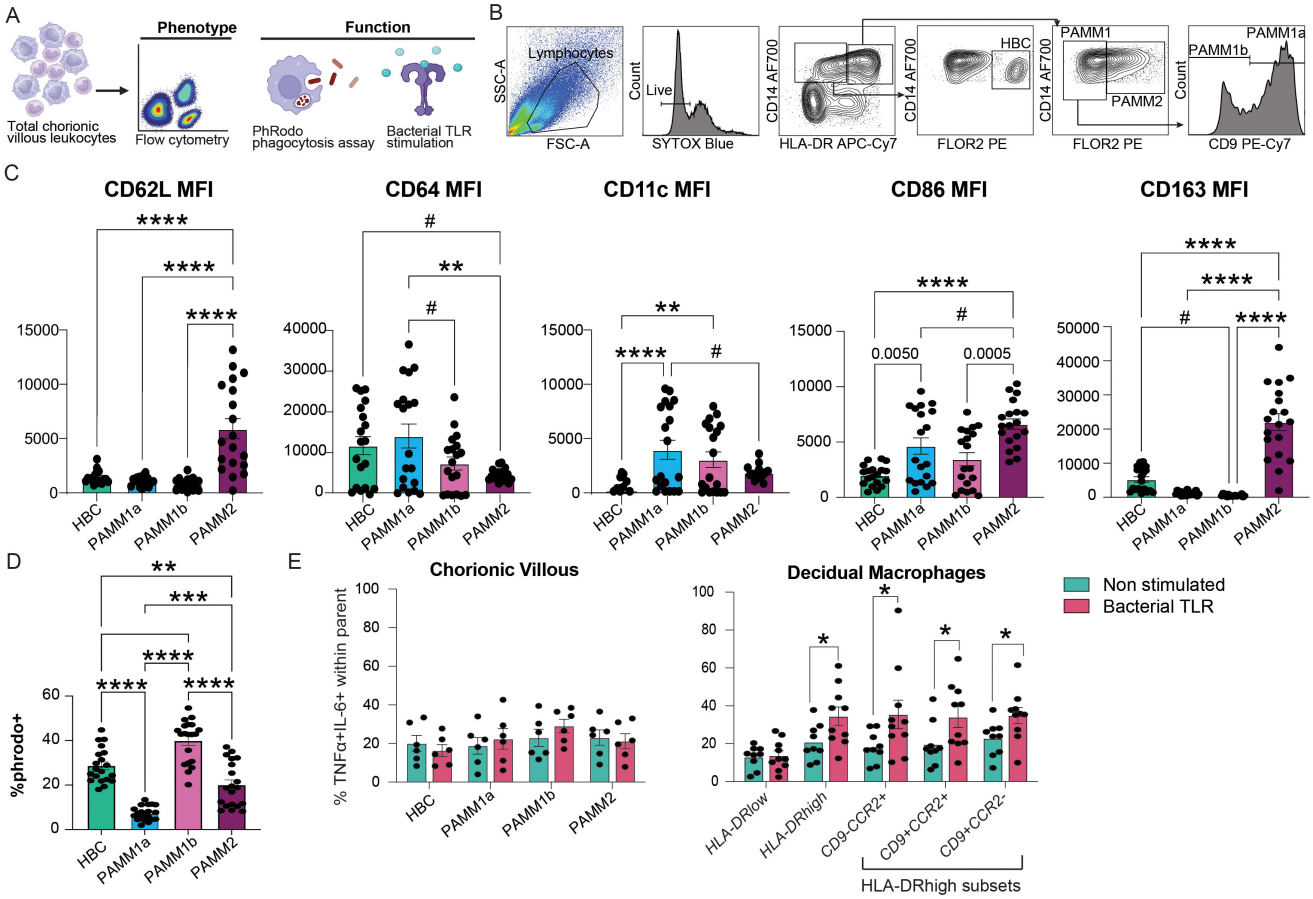
Functional capacity of chorionic villous immune cell subsets at term with pregravid obesity. **(A)** Experimental design – total chorionic villous leukocytes were assayed for the expression of activation markers, phagocytic capacity, and response to bacterial TLR ligands using flow cytometry. **(B)** Gating strategy used for the identification of chorionic villous leukocyte populations by flow cytometry. **(C)** Bar plots of mean fluorescence intensity (MFI) of CD62L (SELL), CD64, CD11c, CD86, and CD163 expression by resting chorionic villous leukocyte subsets. **(D)** Bar plot representing the percentage of *E. coli* pHrodo+ cells in each chorionic villous subset. **(E)** Bar plot of fetal villous (left) and maternal decidua (right) leukocyte responses to stimulation with bacterial TLR ligand cocktail measured by %TNFα+IL-6+ (N=6 for chorionic villous, N=9-10 for maternal decidual tissue). (****p<0.0001, ***p<0.001, **p<0.01, *p<0.05, #p<0.1).

To gain a better insight into functional differences in macrophage subsets at term, we phenotyped the term chorionic villous leukocytes using flow cytometry (N=19) (**Figures 3A, B**). PAMM2 cells highly expressed proteins important for immune surveillance (CD62L) characteristic of monocyte-derived macrophages as well as proteins important for immune signaling and regulation (CD86 and CD163) (**Figure 3C**). HBC and PAMM1a expressed modestly higher expression of CD64 (FcγR-1a), important for innate effector functions, relative to PAMM1b and PAMM2 (**Figure 3C**). Given that phagocytosis is a critical function of placental macrophages (50), we measured the ability of macrophage subsets to internalize fluorescently labeled *E. coli* bioparticles (N=20) by flow cytometry across the macrophage subsets in T3 chorionic villous (**Figure 3D**). HBC and PAMM1b subsets exhibited significantly higher phagocytic capacity than PAMM1a and PAMM2 subsets (**Figure 3D**), highlighting their importance in placental maintenance and antimicrobial surveillance at term. Next, we measured the *ex vivo* response of chorionic villous myeloid cells to bacterial stimulation. Interestingly, the HBC and PAMM subsets did not respond to stimulation as robustly as HLA-DRhigh macrophage subsets within the maternal decidua compartment (**Figure 3E**, **Supplementary Figure 2A**). These findings suggest reprogramming of macrophages in the fetal compartment and emphasize the role of PAMM subsets in tissue homeostasis nearing labor and parturition rather than anti-microbial responses.

### Impact of maternal obesity on chorionic villous immune cell characteristics at term

We next investigated the impact of pregravid obesity on macrophage phenotypes in term chorionic villi (**Figure 1A**). The CCR2^+/-^ scRNA seq data set included subjects who were lean and with obesity (n=4/group). In addition to CD14 and CCR2 antibodies used for FACS sorting (**Supplementary Figure 2B**), cells were also simultaneously stained with a cocktail of oligo-tagged antibodies (CD9, FOLR2, HLA-DR, and CD62L) for parallel assessment of surface protein and RNA expression (**Supplementary Figures 2C, D**). We confirmed a strong overlap between datasets from lean and obese donors (**Supplementary Figure 2E**).

**Figure 4:**
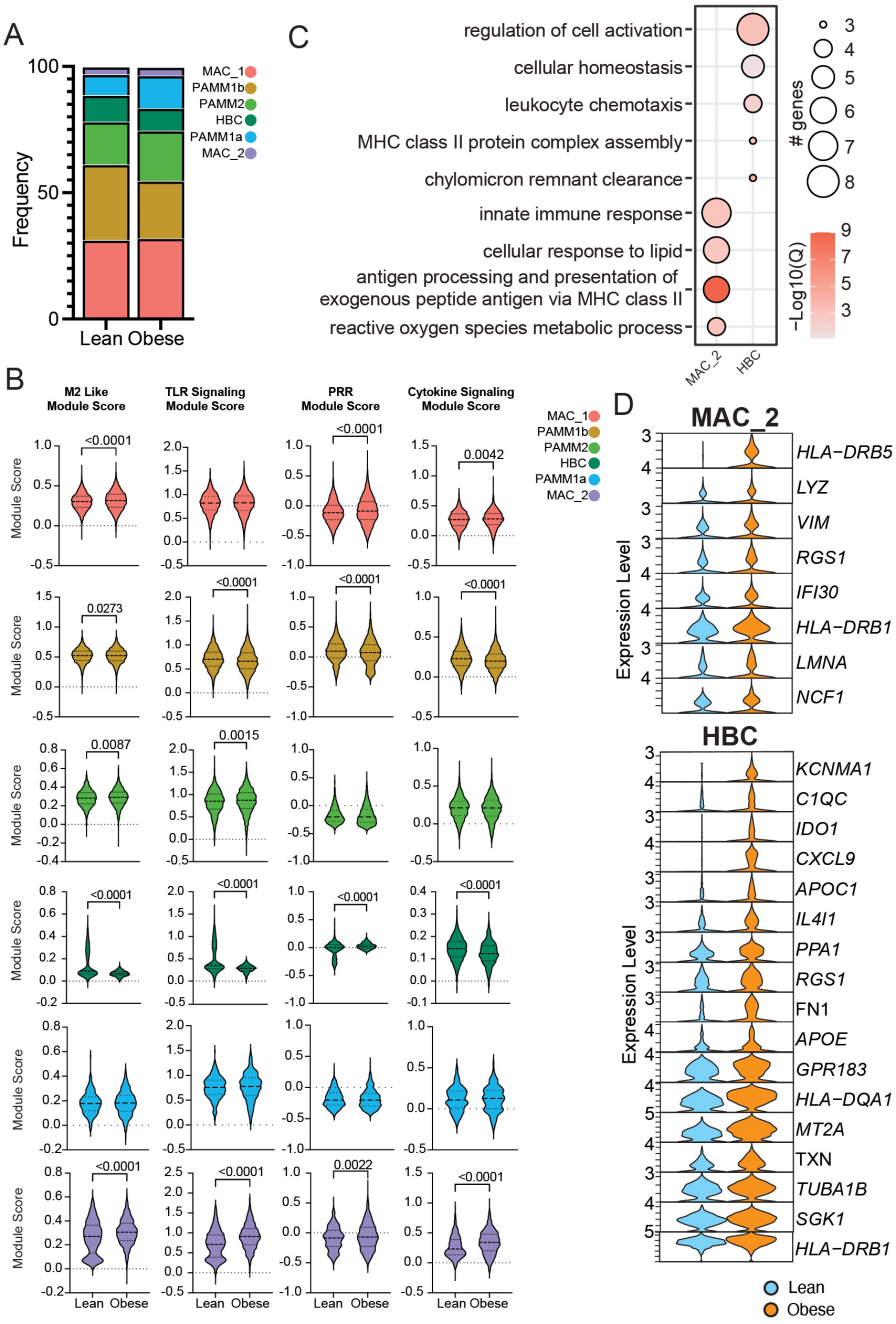
Impact of obesity on the transcriptional profile of term chorionic villous cellular subsets. **(A)** Stacked bar graph of chorionic villous cluster frequencies between groups (lean: 10,647 cells and obese: 13,087 cells). **(B)** Module scores for the terms indicated between lean and obese groups. **(C)** Bubble plot of select gene ontology (GO) terms for DEGs upregulated with obesity in the MAC_2 and HBC clusters. The size of the bubble denotes the number of genes mapping to each GO term, and the intensity of color denotes the -log10(Q-value). **(D)** Violin plots of select DEGs from MAC_2 and HBC subsets.

Our results suggest a slight decrease in the relative abundance of HBC and PAMM1b clusters with concomitant expansions of MAC_2 and PAMM2 clusters with pregravid obesity (**Figure 4A**). Module scores for M2-like features, TLR, PRR, and cytokine signaling were increased in PAMM2, MAC_1, and MAC_2 clusters and decreased in HBC and PAMM1b clusters with pregravid obesity (**Figure 4B**). Differential gene expression revealed that genes associated with innate immune responses (*LYZ*), antigen processing and presentation (*HLA-DRB1, HLA-DRB5, IFI30*), as well as cell signaling and migration (*VIM, RGS1, NCF*) were upregulated in the HBC subset with pregravid obesity (**Figures 4C, D**). On the other hand, genes important in complement activation (*C1QB*), cellular response to oxidative stress (*MT2A, TXN, SGK1*), lipid transport (*APOE, CH25H*), antigen presentation (*HLA-DQA1*, HLADRB1), and regulation of innate immune processes (*KCNMA1, APOC1*, *IDO1*) were upregulated in MAC_2 with pregravid obesity (**Figures 4C, D**).

**Figure 5:**
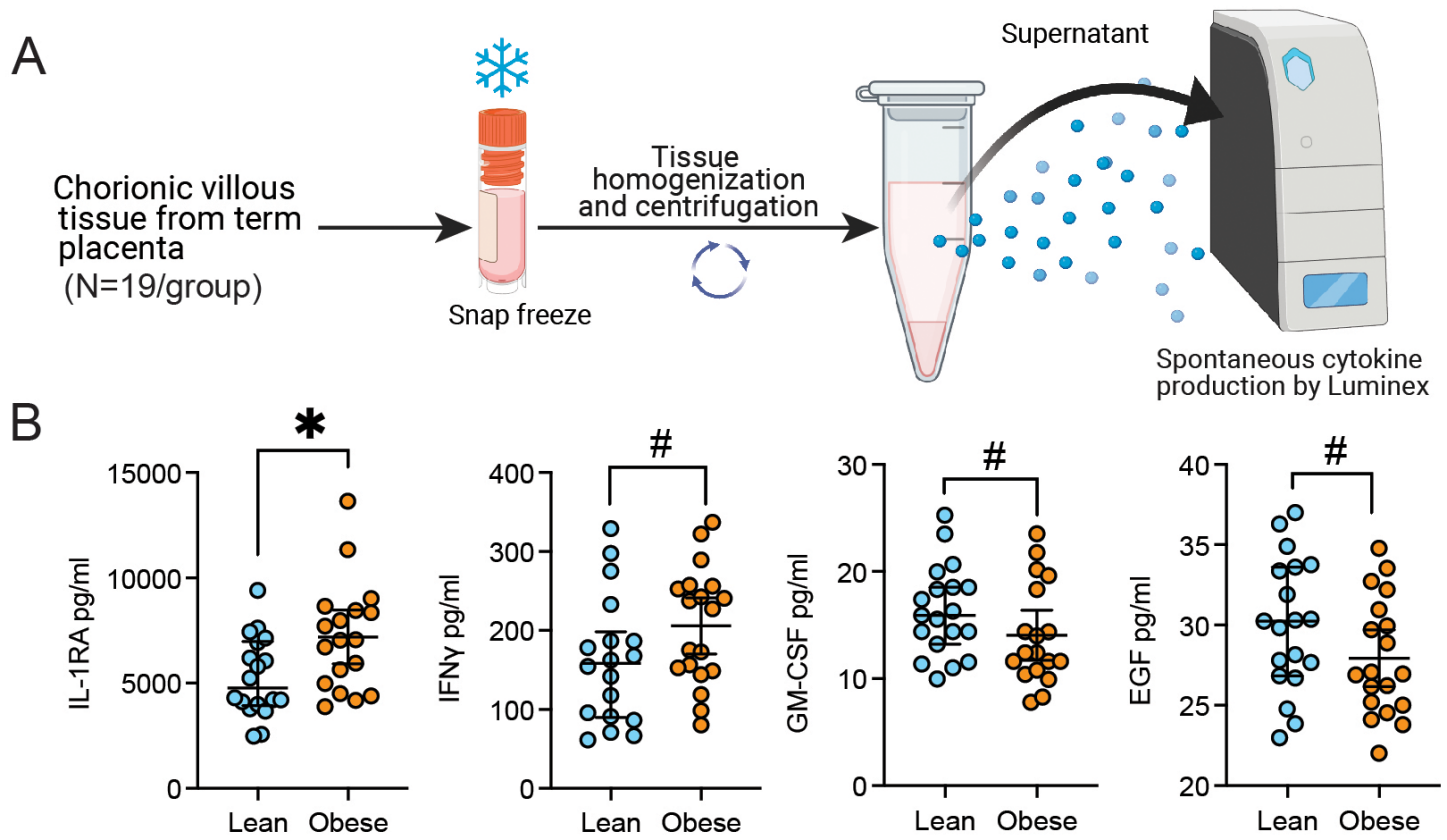
Chorionic villous cytokine and growth factor production with pregravid obesity. **(A)** Experimental design for chorionic villous tissue homogenate Luminex assay. **(B)** Scatter plot of the concentration of select cytokines and growth factors detected in the supernatant of chorionic villous tissue homogenate with or without pregravid obesity (N=19/group). (*p<0.05, #p<0.1).

The concomitant increase in pathways associated with both M-2 and M1-like responses highlight a potential compensatory mechanism in response to low grade chronic inflammation induced by maternal obesity. To explore this concept further, we assayed a panel of cytokines in the supernatant of total chorionic villous tissue homogenate by Luminex (**Figures 1A**, **5A, B**). Levels of IL-1RA and IFNγ were elevated with pregravid obesity, whereas GM-CSF and EGF concentrations were decreased (**Figure 5B**).

### Epigenetic characterization of term chorionic villous immune cells

**Figure 6:**
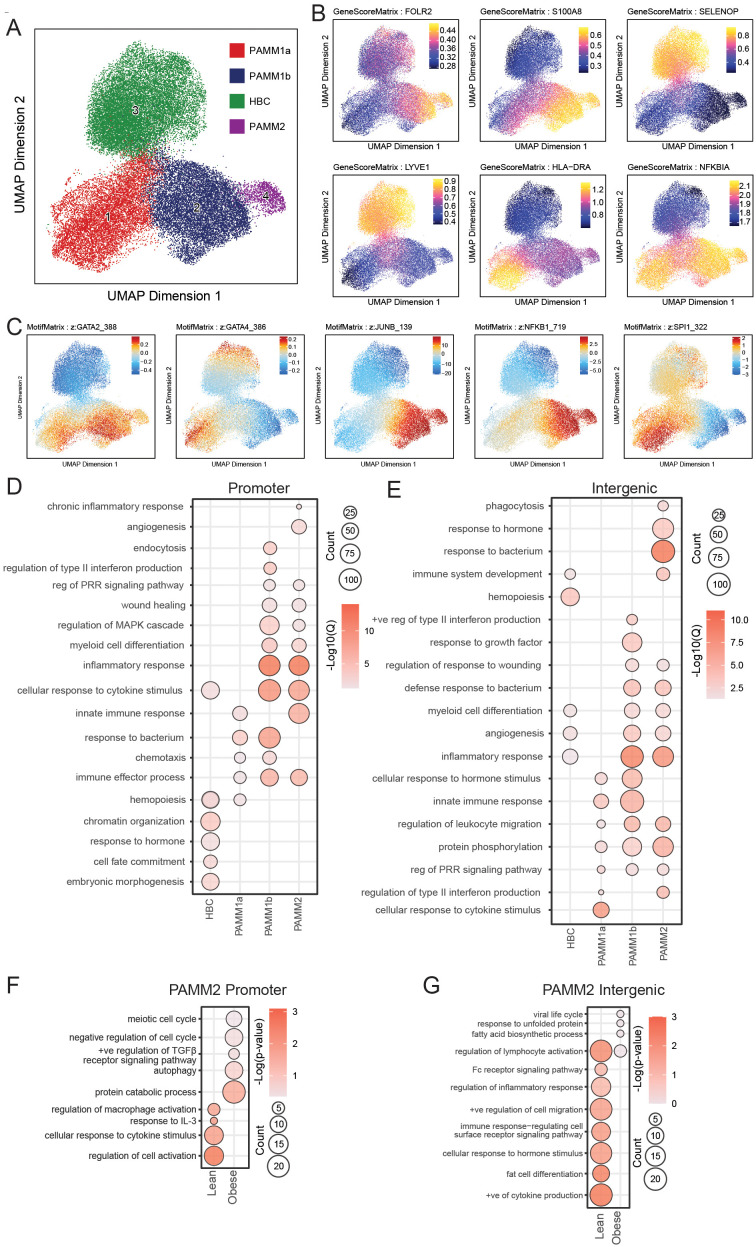
Comparison of the epigenetic regulation of term chorionic villous myeloid subsets by snATACseq. **(A)** UMAP projection of cell subsets within the term chorionic villous (37,316 cells). **(B)** Gene scores and **(C)** Motif Matrix for key transcription factors. **(D, E)** Bubble plot of select GO terms within each subset for **(D)** promoter and **(E)** intergenic regions. The size of the bubble denotes the number of genes mapping to each gene ontology (GO) term, and the intensity of color denotes the -log10(Qvalue). **(F, G)** Bubble plot of select GO terms within the PAMM2 subset between lean and obese groups for **(F)** promoter and **(G)** intergenic regions. The size of the bubble denotes the number of genes mapping to each gene ontology (GO) term, and the intensity of color denotes the -log10(Qvalue).

To uncover the molecular basis underlying transcriptionally diverse macrophage subsets in chorionic villi, we profiled the chromatin accessibility of FACS-purified HBC, PAMM1a, PAMM1b, and PAMM2 cells at T3 (**Supplementary Figure 1A**, 3A) (N=8) using single-nuclei transposase-accessible chromatin sequencing (snATAC-seq) (**Figure 6A**). The identity of the 4 clusters was confirmed based on the chromatin accessibility of canonical marker genes *FOLR2*, *S100A8*, *SELENOP*, *LYVE, NFKIBA*, and *HLA-DRA* (**Figure 6B**, **Supplementary Figure 3B**). We additionally profiled the availability of predicted binding sites for key transcription factors (**Figure 6C**). Binding sites for GATA4 and SPI1 (PU.1) were highly accessible in HBC and PAMM1a subsets (**Figure 6C**), in line with their role in hemopoiesis and trophoblast development (51–54). Accessibility of GATA2, JUNB and NFKB1 binding sites was high in PAMM1b and PAMM2 subsets (**Figure 6C**), in line with their proposed role in inflammatory and anti-bacterial responses.

Functional enrichment of genes with open promoter regions in HBC mapped to embryonic morphogenesis (*FOXF1*, *HOXB5*, *ITGB5*), cell fate commitment (*APC, DLX2*, *FGF13*), and chromatin organization (*MTA3*, *NSD1*, *SMYD3*). Within PAMM1b, genes uniquely mapped to interferon (*IRF8, LGALS9, TLR3*) and endocytosis (*BIN1, ANK2, FCER1G*) processes. In contrast, open promoters in PAMM1b and PAMM1a regulated genes important for chemotaxis (*CCR5*, *CCL3L1*, *S100A9*) and response to bacterium (*C3, IL1A, TNF*). Accessible promoters within PAMM1b and PAMM2 regulated inflammatory response (*BCL6, CXCL2, IL1R1*), MAPK signaling cascade (*IKBKB, MAP3K4, MAPK3*), wound healing (*CDKN1A, CTSG, EREG*), and pattern recognition receptor (PRR) signaling (*IRF7, TLR5, AIM2*) (**Figure 6D**). Finally, accessible promoters in PAMM2 uniquely regulated genes playing a role in angiogenesis (*FN1, PDGFRB, MYDGF*) (**Figure 6D**).

Genes regulated by open intergenic regions in HBC uniquely mapped to hemopoiesis (*PDGFRB, SETD2, MYDGF*) while intergenic regions within all PAMM subsets mapped to PRR signaling (*NOD2, NFKBIZ, IRGM*), protein phosphorylation (*ERBB4, MAP3K21, MYLK3*), and leukocyte migration (*CCR5, ICAM1, SELL*). Furthermore, genes regulated by accessible intergenic regions within HBC, PAMM1b, and PAMM2 mapped to inflammation (*IRGM, C3, CCR5*), angiogenesis (*EPHA, VEGFB, FGF18)*, and myeloid cell differentiation (*TRIB1, TOB2, ZNF16*). Accessible intergenic regions within PAMM1b uniquely mapped to response to type II interferon (*HLA-DPA1, HLA-DPB1, PDE4D*) processes, while those in PAMM2 uniquely mapped to phagocytosis (*LYST, CLN3, DNM2*) and antimicrobial responses (*CAMP, FOS, IL1B*) (**Figure 6E**). Furthermore, pileups of open promoter and intergenic regions revealed unique profiles such as diminished *CXCL12* accessibility in PAMM1b; reduced accessibility of *CCR5, CCR2, FN1, STAT4, VIM, CCL20, CD163*, and increased accessibility of *JAM3* and *HIF3A* in HBC (**Supplementary Figures 3C, D**). Overall, these findings provide novel characterization of the epigenetic profiles of human chorionic villous immune cell subsets at term.

### Chromatin landscape of term chorionic villous immune cells with maternal obesity

To determine the impact of maternal obesity on the chromatin accessibility of term chorionic villous immune cells, we further stratified our snATAC-seq findings by maternal BMI (N=4/group). Chromatin accessibility differences were only detected in the PAMM2 subset (**Figures 6F, G**). Genes regulated by promoter regions open only in the lean group uniquely mapped to regulation of cell activation (*MMP8*, *TNIP2*, *SRC*), and responses to IL-3 (*IL3RA*, *MT2A*, *SH2B3*) and other cytokine stimulus (*MPAK13*, *CCL7*, *MT2A*). Similarly, intergenic regions unique to the lean group mapped to cytokine production (*FGR*, *HGF*, *PIK3R1*), regulation of inflammatory responses (*SYK*, *TNFSF11*, *NLRP3*), and Fc-receptor signaling (*PIGR*, *FCMR*). However, with obesity, we observed increased accessibility of genes involved in cell cycle regulation (*ATM*, *CDKN1C*, *BCL2L11*), autophagy (*HSPA8*, *IRF8*, *PEX5*), and protein catabolism (*CDC34*, *CTSB*, *UBB*) (**Figure 6F**). Intergenic regions uniquely accessible in the obese group mapped to viral life cycle (*KPNA3*, *PCSK5*, *SLC52A1*), unfolded protein response (*HSPD1*, *HSPB1*, *HERPUD1*), and fatty acid biosynthesis (*LIPG*, *TECR*, *ELOVL3*) (**Figure 6G**). Considering that PAMM2 cells originate from early uterine tissues and are likely contaminants from the maternal compartment, these findings align with our previous reports of maternal immune dysregulation in decidual tissues with maternal obesity (46).

### Altered UCB-derived microglia function with pregravid obesity

**Figure 7:**
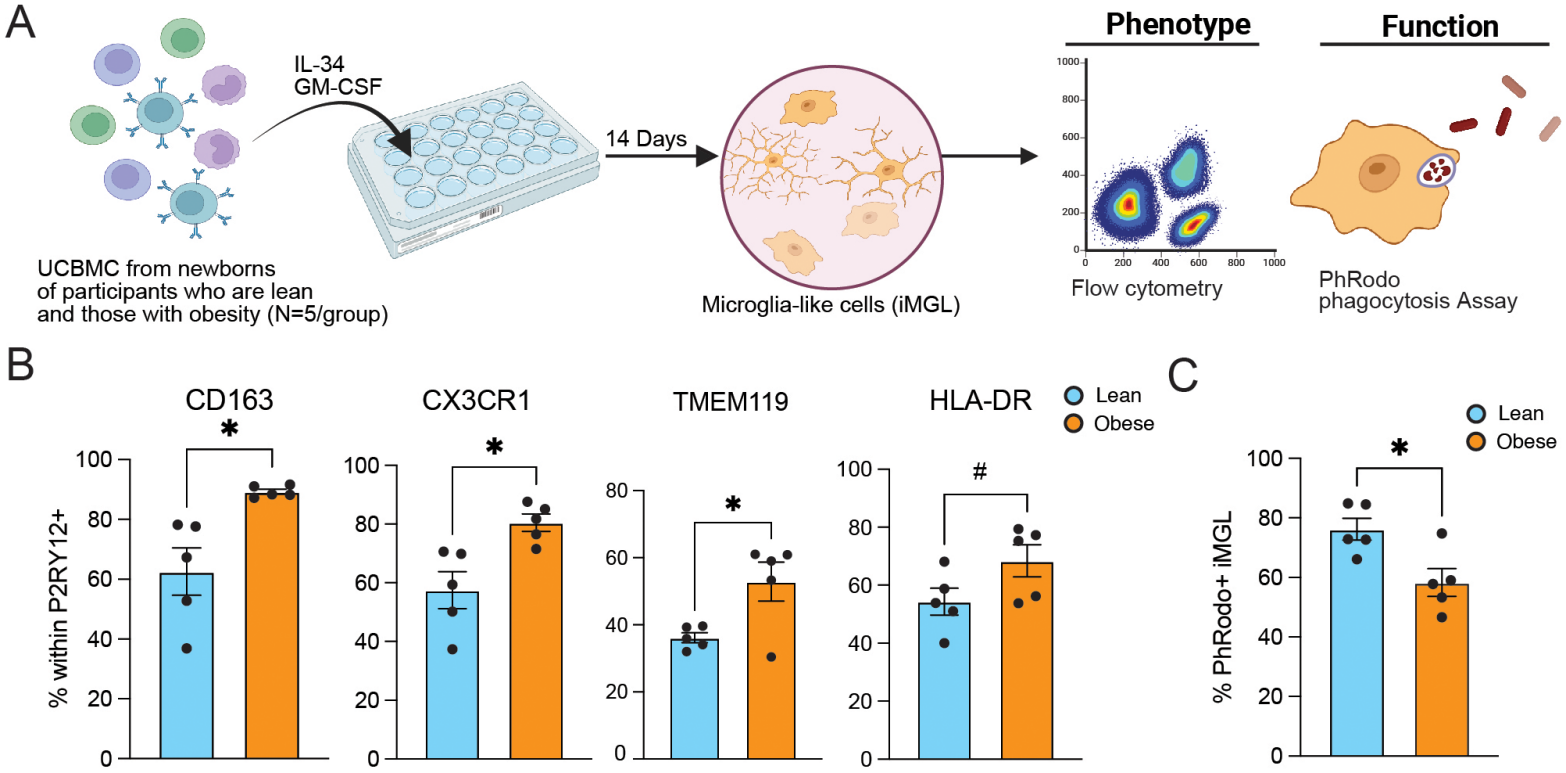
Impact of pregravid obesity on UCB-derived microglia-like cell phenotype and function. **(A)** Experimental design for the derivation of microglia-like cells (iMGL) from umbilical cord blood mononuclear cells (UCBMC) of newborns of lean subjects and those with obesity. The expression of activation markers and phagocytic capacity of iMGLs were then assayed using flow cytometry. **(B, C)** Bar plots of **(B)** the percent of CD163, CX3CR1, TMEM119, and HLA-DR **(C)** percentage of *E. coli* pHrodo+ cells expressed by P2RY12+ iMGL in lean and obese groups (N=5/group). (*p<0.05, #p<0.1).

Several studies have reported an increased prevalence of neurobehavioral dysregulation in children exposed to maternal obesity *in utero* (28, 49, 55, 56), possibly driven by abnormal activation of microglia which can negatively impact synaptic pruning and regulation of neurogenesis (51). Since HBCs share fetal yolk-sac origins with microglia, the heightened expression of innate immune pathways observed in this subset may indicate dysregulated microglia development. To test this hypothesis, we leveraged an *in vitro* model of microglia derived from umbilical cord blood (UCB) (45) (n=5/group) (**Figures 1A**, **7A**). Induced UCB-derived microglia-like cells (iMGL) expressed higher levels of activation markers CD163, CX3CR1, TMEM119, and HLA-DR (**Figure 7B**) but reduced phagocytic capacity with maternal pregravid obesity (**Figure 7C**). Since phagocytosis of myelin fragments and debris clearance in neuron maintenance is one of the primary functions of microglia (57, 58), these findings provide a potential explanation for the observed increased rates of intellectual disability and poor cognitive performance and motor skills in offspring exposed to maternal obesity *in utero* (28, 49, 56).

## Discussion

Tissue-resident macrophages of many adult organs originate in the fetal yolk sac (59–61). As such, programming of the fetal immune system begins before birth and can be influenced by the changes in maternal environment, such as obesity (62). The fetal placenta and villous structures within develop during early pregnancy, and by 10 weeks of gestation, a heterogeneous population of fetal-yolk-sac-derived macrophages (HBCs) seed the developing villous tree, where they play a critical role in placental development and antimicrobial responses throughout gestation (63, 64). Simultaneously, yolk-sac progenitors that seed in the fetal brain mature into microglia, the immune sentinels of the brain (61, 65). Given their common yolk-sac origin, emerging studies have correlated HBC function with other tissue-resident macrophages, such as microglia, making fetal placental tissues (chorionic villous) an accessible model of early immune programming and neurodevelopment (10, 11). However, there is a paucity of human studies that address the longitudinal changes in HBC populations with gestational age and in the context of maternal obesity (15). Additionally, a diverse population of placenta-associated maternal monocytes and macrophages (PAMM) were recently identified in the first-trimester placenta that invades the villous tissue compartment from maternal tissues to aid placental repair and debris clearance (5, 66). Other groups have also described monocytes and macrophages of maternal origin within the chorionic villous compartment (4, 5, 7, 8, 42); however, a consensus on the classification of these subsets is lacking (4, 5, 7, 8, 42). Therefore, an atlas of human chorionic villous immune landscape at term and the impact of pregravid obesity on these immune cells are needed.

Here, we profiled the immune compartment of term villous tissue isolated from healthy pregnant persons undergoing scheduled cesarean delivery to generate a detailed map of transcriptomic, phenotypic, and functional diversity of term HBC and PAMM subsets. We further provide novel insight towards the epigenetic profiles of term chorionic villi macrophage subsets. Next, given the strong association between maternal pre-pregnancy BMI and pregnancy complications (19–21, 23, 26, 32, 65, 67–77), we investigated the impact of pregravid obesity on the functional rewiring of chorionic villous immune cells. Finally, we leveraged an established model of microglia-like cells (iMGL) derived from umbilical cord blood mononuclear cells (UCBMC) to model poor neurodevelopment in offspring with *in-utero* exposure to inflammation due to maternal factors such as obesity (10, 11).

Immune cell populations of the first trimester chorionic villous are predominantly composed of HBCs (5, 8). We report a reduction in the relative abundance of HBCs with gestational age. Interestingly, our analysis also revealed a shift in their gene expression program towards immune activation and cytokine signaling at T3, possibly in preparation for labor and parturition. Indeed, the accumulation of inflammatory, M1-like phenotype of HBCs has been reported to play an essential role in promoting uterine contraction nearing parturition (40, 50, 78, 79). Consistent with these reports, our results demonstrated high phagocytic capacity in T3 HBCs relative to PAMM1a and PAMM2, although lower levels relative to PAMM1b subsets. This differs from earlier reports of higher levels of phagocytosis in PAMM1a relative to HBC in T1 (5, 6) and indicates an evolution of HBC and PAMM1a with gestational age.

Previous studies have reported that, in T1, PAMM1a cells are morphologically similar to macrophages with high lipid content, suggesting their likely function in the clearance of cellular debris and repair of the syncytium following damage (5, 6). In line with those prior studies, our results show that the marker genes that define this subset enriched to “cellular response to lipids”. However, our results also indicate a shift from immune resolving (down-regulation of genes with a role in wound healing) to immune activation (increased expression of genes important for pathogen sensing and TLR signaling) with gestation. These results suggest a shift in PAMM1a function towards microbial surveillance mechanisms that may be exhausted in late pregnancy. PAMM1b cells have been reported to be morphologically similar to maternal-derived circulating classical monocytes and as possible precursors to PAMM1a macrophages (5). Our results indicate that wound healing and migration module scores are decreased in PAMM1b cells in late pregnancy while phagocytosis capacity increases, indicating that these cells remain important for tissue homeostasis and remodeling in late pregnancy.

Infiltrating maternal macrophages, PAMM2 cells, are found in relatively low abundance in fetal villous tissues at T1 (6). Our results show a higher abundance of PAMM2 at T3 that harbor transcriptional signatures associated with elevated TLR/chemokine signaling. Additionally, the PAMM2 subset expresses higher levels of regulatory and homing marker CD62L, activation marker CD86, and M2-like macrophage marker CD163, highlighting the complexity of placental macrophage populations. Finally, we identified two additional macrophage subsets at T3. Relative abundance of MAC_1 increases at T3 while their transcriptional profile shifts from antimicrobial responses in T1 to protein folding in T3, possibly indicating a more regulatory phenotype in late pregnancy. On the other hand, MAC_2 cells slightly decrease with gestational age, but express higher levels of genes important for antimicrobial responses, suggesting an increasing role for this infiltrating macrophage subset in preventing infection in late pregnancy (80).

Chronic low-grade inflammation secondary to maternal obesity is the likely underlying cause of obesity-related obstetric and perinatal complications (81, 82). Indeed, the release of pro-inflammatory cytokines such as IL-6 and TNFα in response to maternal inflammation can readily enter fetal circulation and impact fetal development (83). Here, we report an increase in IL-1RA and IFNγ in villous tissue homogenate with maternal obesity, possibly attributed to increased Th1 cytokine secretion (IL-1, TNFα, IFNγ) by infiltrating maternal macrophages (PAMM) with maternal obesity (84). Additionally, previous studies have shown an increased abundance and altered morphology of HBCs (38) and increased infiltration of maternal macrophages into the chorionic villous with pregravid obesity (32, 69). Indeed, high levels of infiltrating maternal macrophages are a hallmark of chorionic villous inflammatory abnormalities (83, 85). Here, we report a slight decrease in the abundance of HBC that is accompanied by an increase in infiltrating PAMM2 and MAC_2 cells. Furthermore, module scores for PRR signaling were reduced with pregravid obesity across multiple subsets. This observation is in line with our previous studies that decidual macrophages (46), peripheral monocytes (49), and cord blood monocytes (86, 87) adapt a tolerant-like phenotype with maternal obesity.

The impact of maternal obesity on HBCs may provide insight towards disruptions in neurodevelopment in the offspring, given their shared fetal yolk sac origin with brain resident macrophages called microglia (10). A recent study has shown that rewiring of fetal placental macrophages is linked to hyperinflammatory microglia and poor brain development in the offspring of mice fed a high-fat diet (9). Here, using a model of microglia derived from UCBMC *in vitro* (iMGL), we report an increase in the expression of activation markers (CD163, CX3CR1, TMEM119, HLA-DR) with pregravid obesity, while phagocytic capacity was reduced. Recent studies have shown that increased early microglia activation decreases the neural precursor cell pool, alters synaptic density, and delays neuronal circuit maturation (88). Furthermore, microglia guide normal brain development through phagocytosis of dying cells and perturbations leads to altered synaptic pruning (89). Our results shed light on possible mechanisms underlying poor cognitive performance and increased rates of neurodevelopment disorders in the offspring of women with obesity.

Epigenetic regulation of the placenta is important in determining placental function and fetal programming (90, 91). However, studies have yet to define the chromatin accessibility profile of immune cells within the chorionic villous. At term, we report accessible promoter and intergenic regions that regulate inflammatory responses by snATAC-seq. Open regions within HBCs mapped to hemopoiesis, embryonic development, hormone responses, and immune system development pathways reflecting the yolk-sac origin of HBCs. Additionally, regions accessible in HBCs contained binding sites for the transcription factor SPI1 (PU.1). This is in line with previous studies in murine models that show that HBCs are highly dependent on PU.1, as its loss of function leads to poor placental remodeling and angiogenesis (92). Accessibility to binding sites for SPI1 (PU.1) as well as GATA4 were also evident in the PAMM1a cluster. This observation is aligned with the fact that marker genes within PAMM1a enriched to GO terms associated with hemopoiesis. Furthermore, binding sites for transcription factors GATA2 and NF-κB were accessible across PAMM subsets, highlighting their role in regulating inflammation (90, 93) and antimicrobial responses (94). Binding sites for JUNB, which plays a critical role in placental vascularization (95), were detected in PAMM1b and PAMM2.

Given that pregravid obesity alters the programming of fetal immune cells *in utero*, we further stratified our snATAC-seq findings by maternal BMI. We report significant differences in the epigenetic profile of PAMM2 cells with pregravid obesity. Previous studies have shown an immunotolerant-like phenotype characterized by decreased chromatin accessibility at inflammatory loci and dampened antimicrobial responses in maternal monocytes (48, 96, 97). Here, genes regulated by open promoter and intergenic regions accessible in the lean group uniquely mapped to functions important for cell activation and inflammatory responses, while those in the obese group mapped to cell cycle regulation and maintenance of oxidative stress. These results suggest that this immune-tolerant phenotype is possibly maintained by immune cells of maternal origin in the fetal placenta with maternal obesity.

In summary, our data provide an atlas of villous myeloid cells with gestational age. We show that chorionic villous immune cells at term exhibit a heightened inflammatory state relative to the first trimester, likely to support labor and parturition. Notably, PAMM1b monocytes and HBCs exhibit the most phagocytic capacity of the identified subsets, while PAMM2 were most activated. Additionally, we reveal novel differences in chromatin accessibility between chorionic villous myeloid subsets. Pregravid obesity led to increased expression of inflammatory genes, particularly among HBC and PAMM1a subsets, but dampened expression of antimicrobial genes, further reflected by a lack of responses by villous myeloid cells to stimulation by bacterial ligands. Finally, we highlight the shared yolk-sac origin of HBCs and microglia, providing insight towards *in utero* immune programming by pregravid obesity and poor neurodevelopmental outcomes. Altogether, our study highlights chorionic villous myeloid cell diversity with gestational age, profiles the functional and epigenetic landscape of term chorionic villous macrophage subsets, and emphasizes the impact of maternal environmental exposures, such as obesity, on fetal immune programming. Finally, our study provides the groundwork for term villous immune profiles and future studies could further characterize the spatial and functional landscape of term villous immune cells.

## Data Availability

The data presented in the study are deposited in the NCBI Sequence Read Archive repository, accession number PRJNA970789 (CD45+ scRNAseq) and PRJNA1093455 (ATAC- and CITE-seq).

## Supplementary material

Supplementary Table 1 Gene markers for clusters identified by 3′ gene expression analysis of total chorionic villous, related to **Figure 1**.

Supplementary Table 2 Module scores for clusters identified by 3′ gene expression analysis, related to **Figures 1**, **2** and **4**.

Supplementary Table 3 Gene markers for clusters identified by Single-nuclei ATAC-Seq data analysis, related to **Figure 7**.

Supplementary Figure 1 Integration of first trimester and term chorionic villous transcriptional profiles. **(A)** Experimental design for scRNA-seq, CITE-seq, and snATAC-seq experiments. Chorionic villous leukocytes were FACS sorted for CD45+ cells and single-cell suspensions were subjected to gene expression profiling using 10x 3’single-cell gene expression protocol (scRNA-seq) (N=4, 2 lean and 2 obese). Additional chorionic villous leukocytes FACS sorted for CCR2+ cells and single-cell suspensions were subjected to transcriptome-based profiling using CITE-seq (N=8, 4 lean and 4 obese). Chorionic villous leukocytes were also subjected to snATAC-seq for chromatin accessibility (N=8, 4 lean and 4 obese). **(B)** UMAP of cell clusters from decidual leukocytes and matched maternal peripheral blood mononucleated cells (PBMC), chorionic villous leukocytes and matched umbilical cord blood mononucleated cells (UCBMC). **(C)** UMAP of cell clusters from previously published first-trimester placenta (top left) and markers used to isolate known chorionic villi cell populations. **(D)** UMAP of integrated CCR2+/- CITEseq T3 (left), T1 (Vento-Tormo, Nature, 2018, middle), and our CD45+ T3 (right) data.

Supplementary Figure 2 Impact of obesity on the transcriptional profile of chorionic villi cellular subsets. **(A)** Gating strategy used for identification of decidua macrophage populations by flow cytometry. **(B)** Gating strategy used for the sorting of cell population for CD14+CCR2+/- CITEseq. **(C)** Feature plots of expression for the indicate markers from RNA (left) or protein (right, CITEseq) used for cluster identification. **(D)** Violin plots of the expression of select oligo-tagged antibodies for RNA and TotalSeqB **(E)** UMAP colored by subject phenotype.

Supplementary Figure 3 Comparison of the epigenetic regulation of term chorionic villous myeloid subsets by snATACseq. **(A)** Gating strategy used for the FACS sorting of villous leukocyte populations for snATACseq. **(B)** Violin plots showing the expression of canonical markers from scRNA-seq data supporting the identity of the 4 clusters identified as accessible by snATAC-seq. **(C, D)** Pileups of key cytokines and transcriptions factors for **(C)** promoter and **(D)** intergenic regions.
