# Supplementary figures and images for: The immune landscape of fetal chorionic villous tissue in term placenta
Source: Front Immunol. 2025 Jan 13;15:1506305. doi: 10.3389/fimmu.2024.1506305 (PMC11769816; doi:10.3389/fimmu.2024.1506305)

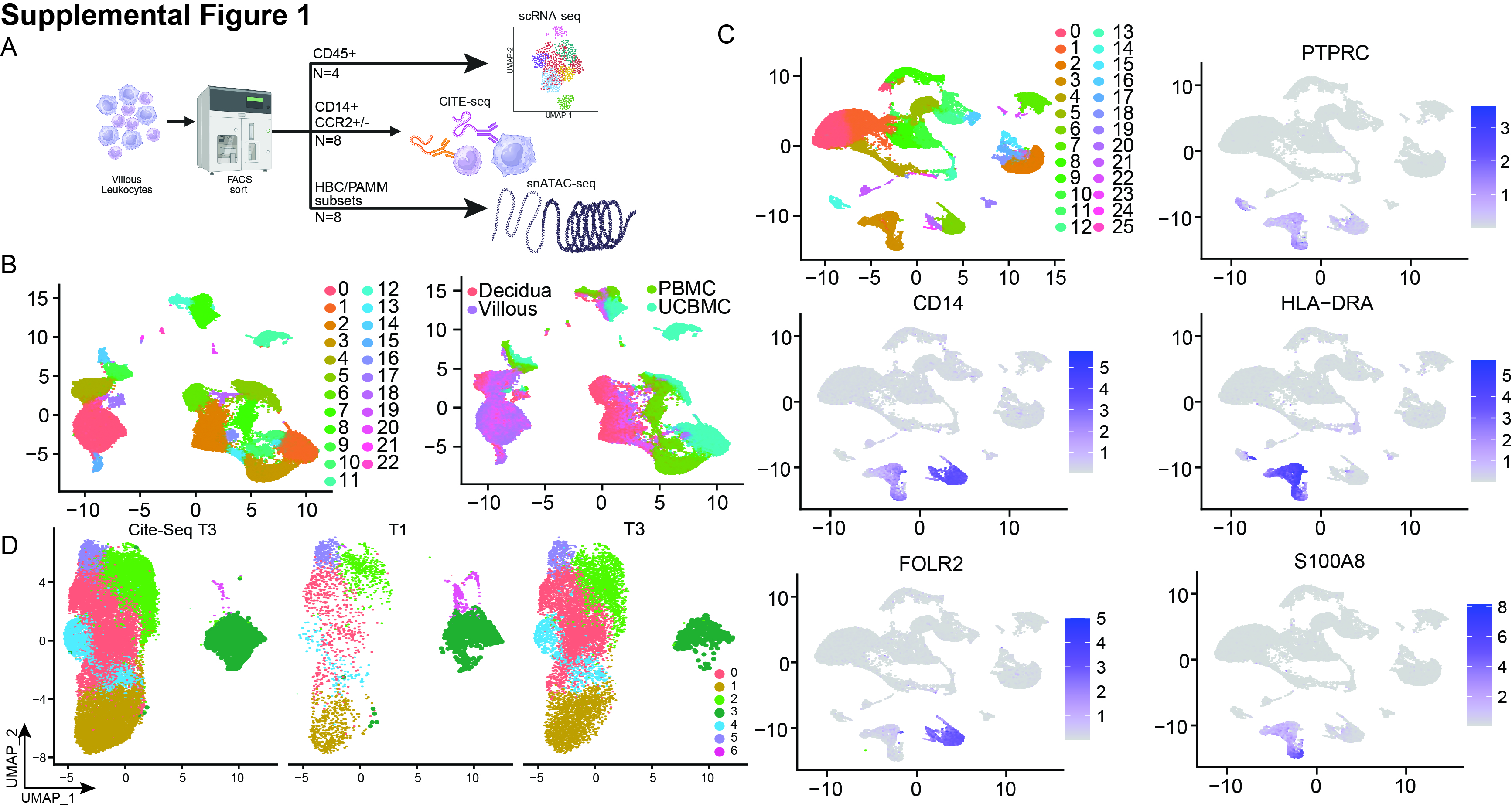

Supplement: Supplementary Figure 1 — Integration of first trimester and term chorionic villous transcriptional profiles. (A) Experimental design for scRNA-seq, CITE-seq, and snATAC-seq experiments. Chorionic villous leukocytes were FACS sorted for CD45+ cells and single-cell suspensions were subjected to gene expression profiling using 10x 3’single-cell gene expression protocol (scRNA-seq) (N=4, 2 lean and 2 obese). Additional chorionic villous leukocytes FACS sorted for CCR2+ cells and single-cell suspensions were subjected to transcriptome-based profiling using CITE-seq (N=8, 4 lean and 4 obese). Chorionic villous leukocytes were also subjected to snATAC-seq for chromatin accessibility (N=8, 4 lean and 4 obese). (B) UMAP of cell clusters from decidual leukocytes and matched maternal peripheral blood mononucleated cells (PBMC), chorionic villous leukocytes and matched umbilical cord blood mononucleated cells (UCBMC). (C) UMAP of cell clusters from previously published first-trimester placenta (top left) and markers used to isolate known chorionic villi cell populations. (D) UMAP of integrated CCR2+/- CITEseq T3 (left), T1 (Vento-Tormo, Nature, 2018, middle), and our CD45+ T3 (right) data. [file Image1.jpeg]

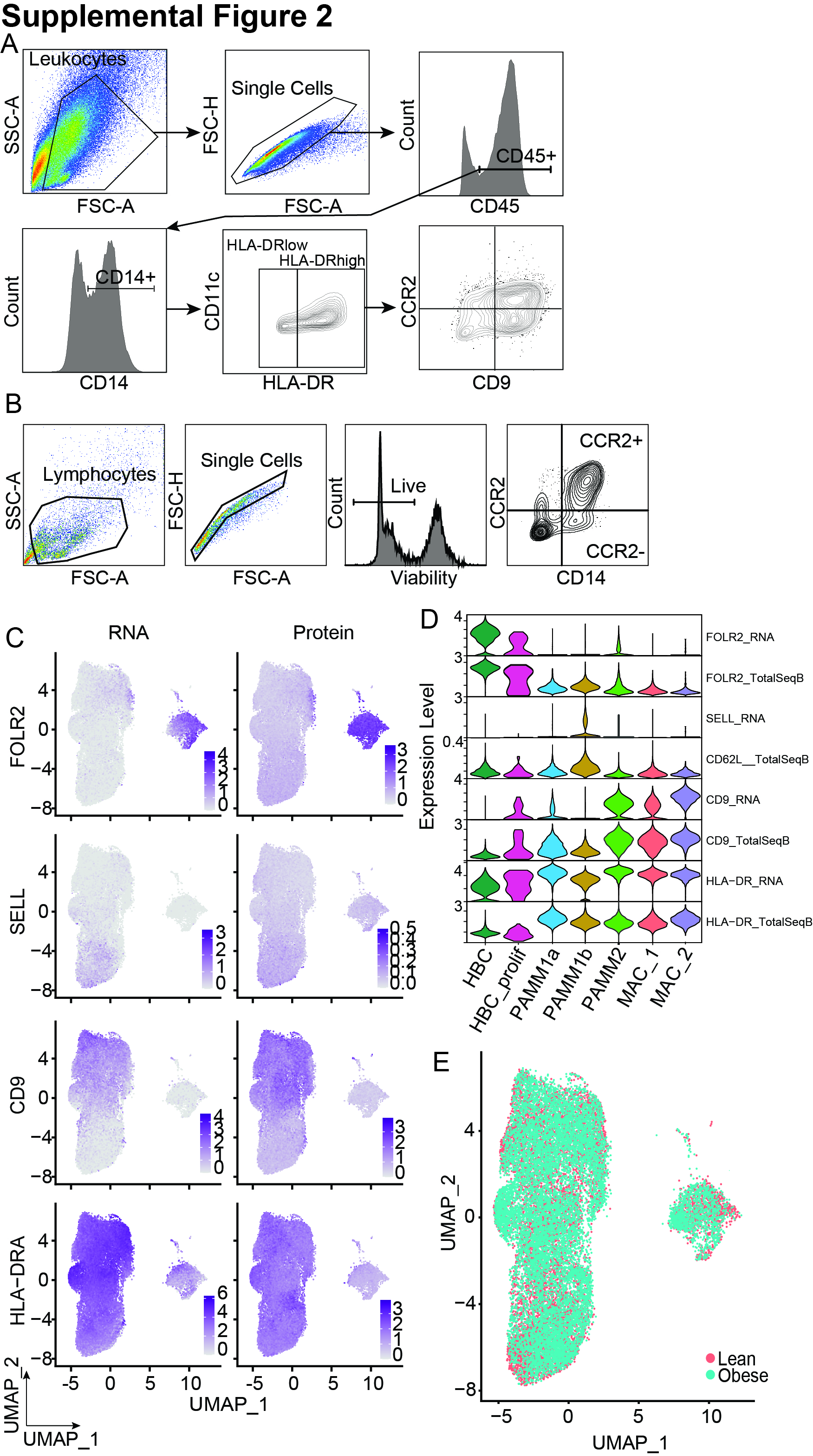

Supplement: Supplementary Figure 2 — Impact of obesity on the transcriptional profile of chorionic villi cellular subsets. (A) Gating strategy used for identification of decidua macrophage populations by flow cytometry. (B) Gating strategy used for the sorting of cell population for CD14+CCR2+/- CITEseq. (C) Feature plots of expression for the indicate markers from RNA (left) or protein (right, CITEseq) used for cluster identification. (D) Violin plots of the expression of select oligo-tagged antibodies for RNA and TotalSeqB (E) UMAP colored by subject phenotype. [file Image2.jpeg]

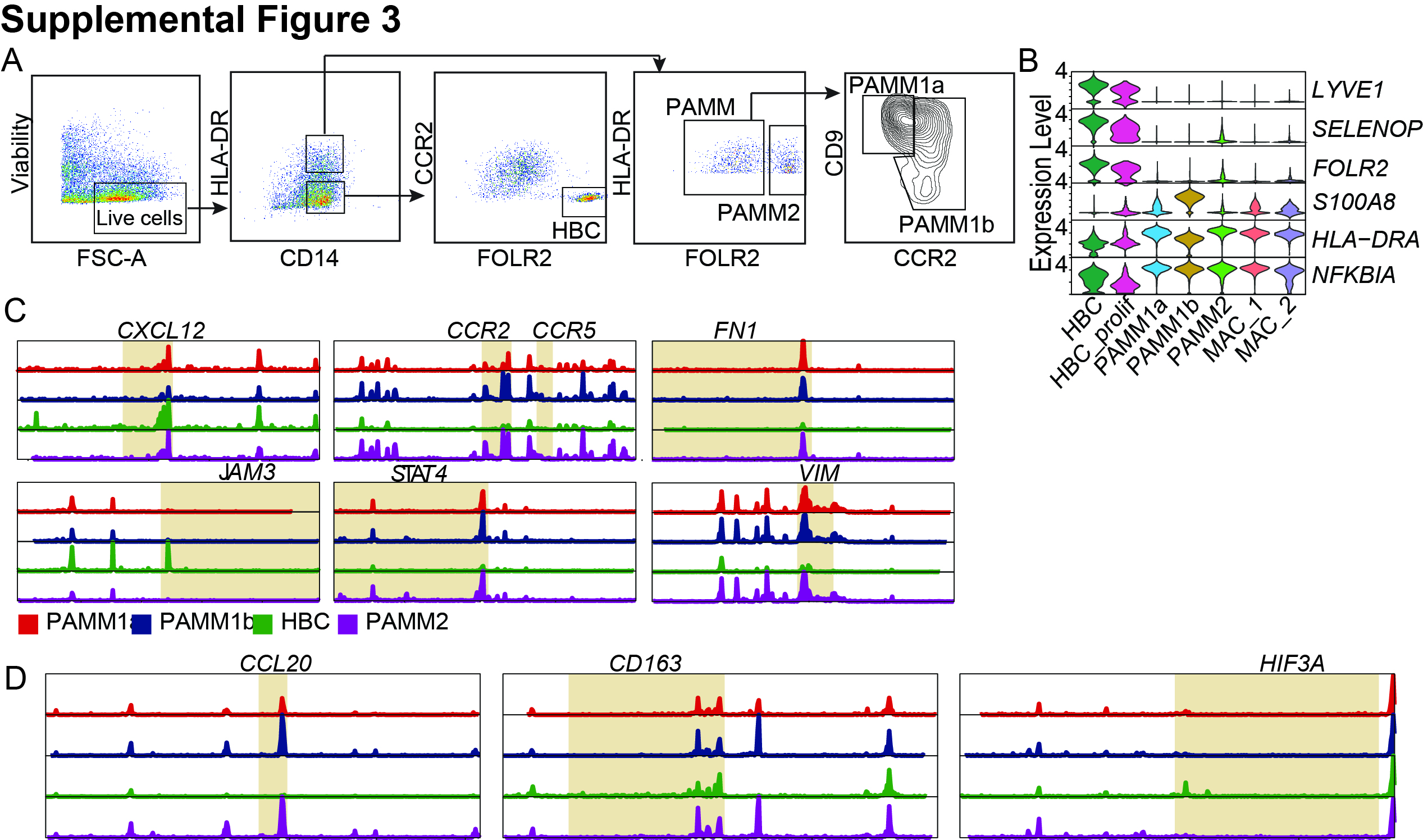

Supplement: Supplementary Figure 3 — Comparison of the epigenetic regulation of term chorionic villous myeloid subsets by snATACseq. (A) Gating strategy used for the FACS sorting of villous leukocyte populations for snATACseq. (B) Violin plots showing the expression of canonical markers from scRNA-seq data supporting the identity of the 4 clusters identified as accessible by snATAC-seq. (C, D) Pileups of key cytokines and transcriptions factors for (C) promoter and (D) intergenic regions. [file Image3.jpeg]
